# A Comparison of Charge Carrier Dynamics in Organic and Perovskite Solar Cells

**DOI:** 10.1002/adma.202101833

**Published:** 2021-11-12

**Authors:** Jiaying Wu, Hyojung Cha, Tian Du, Yifan Dong, Weidong Xu, Chieh‐Ting Lin, James R. Durrant

**Affiliations:** ^1^ Department of Chemistry and Centre for Processable Electronics Imperial College London London W12 0BZ UK; ^2^ Department of Hydrogen & Renewable Energy Kyungpook National University Daegu 41566 South Korea; ^3^ SPECIFIC IKC College of Engineering Swansea University Bay Campus, Fabian Way Swansea Wales SA1 8EN UK

**Keywords:** charge recombination, charge transport, charge trapping, photophysics, solar cells

## Abstract

The charge carrier dynamics in organic solar cells and organic–inorganic hybrid metal halide perovskite solar cells, two leading technologies in thin‐film photovoltaics, are compared. The similarities and differences in charge generation, charge separation, charge transport, charge collection, and charge recombination in these two technologies are discussed, linking these back to the intrinsic material properties of organic and perovskite semiconductors, and how these factors impact on photovoltaic device performance is elucidated. In particular, the impact of exciton binding energy, charge transfer states, bimolecular recombination, charge carrier transport, sub‐bandgap tail states, and surface recombination is evaluated, and the lessons learned from transient optical and optoelectronic measurements are discussed. This perspective thus highlights the key factors limiting device performance and rationalizes similarities and differences in design requirements between organic and perovskite solar cells.

## Graphical abstract

The similarities and differences in the charge carrier dynamics in organic solar cells and organic–inorganic hybrid metal halide perovskite solar cells, two leading technologies in thin‐film photovoltaics, are discussed, linking these back to the intrinsic material properties of organic and perovskite semiconductors, and how these factors impact on photovoltaic device performance is elucidated.

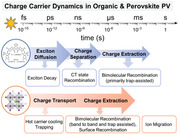

## 1 Introduction

Solution‐processable semiconductors offer the potential for the scalable manufacturing of low‐cost, lightweight, integratable, and flexible photovoltaic devices. In particular, solution‐processed solar cells based on both organic semiconductors and organic–inorganic hybrid metal halide perovskites have recently shown rapid improvements in photovoltaic performance and operation stability.^[^ ^1^, ^2^, ^3^, ^4^, ^5^ ^]^ The power conversion efficiency (PCE) of organic solar cells (OSCs) containing a conjugated polymer donor and a small molecule acceptor has recently surpassed 18%, driven mainly by advances in the design of photoactive materials, thin‐film morphology, and device architecture.^[^ ^6^, ^7^, ^8^, ^9^, ^10^, ^11^, ^12^, ^13^, ^14^ ^]^ In particular, recent advances in the design of nonfullerene electron acceptors (NFAs) have enhanced device performance by broadening light absorption, lowering voltage losses,^[^ ^6^, ^7^, ^8^, ^9^, ^15^ ^]^ and enhancing environmental stability^[^ ^1^, ^10^, ^11^, ^16^ ^]^ compared to conventional fullerene‐based electron acceptors. On the other hand, organic–inorganic hybrid metal halide perovskite solar cells (abbreviated herein to perovskite solar cells or PSCs) show PCEs of over 25%, comparable to single crystalline Si devices,^[^ ^17^ ^]^ driven mainly by optimization of compositional tuning and material processing,^[^ ^18^ ^]^ as well as advances in charge transport layers.^[^ ^19^, ^20^ ^]^ PSCs have been studied in a range of device architectures, with advances in efficiency complemented by advances in stability.^[^ ^21^, ^22^, ^23^ ^]^ Herein for simplicity, we focus on PSCs and OSCs with p–i–n planar device architectures, an architecture widely employed in both technologies. As such these OSCs and PSCs share similarities in sandwich device architecture, fabrication process, and device characteristics, facilitating direct comparisons between these two classes of solution‐processed solar cells.^[^ ^24^, ^25^, ^26^ ^]^

OSCs have been widely studied and consensus reached on many aspects of device function,^[^ ^15^, ^35^, ^36^, ^37^, ^38^, ^39^ ^]^ although recent advances in device efficiency enabled by new NFAs have exceeded previous theoretical predictions,^[^ ^40^ ^]^ and reopened questions of the processes limiting device efficiency. By contrast, PSCs are a relatively new technology, where efficiencies have progressed at remarkable speed, and while rapid advances in understanding have also been made, some aspects of the fundamental processes underlying device function are still emerging. In both OSC and PSC devices, the photophysics and charge carrier dynamics are central to determining how efficiently absorbed light is converted into electrical power, and therefore knowledge of these processes is critical in guiding materials and device design.^[^ ^41^ ^]^

**Figure 1:**
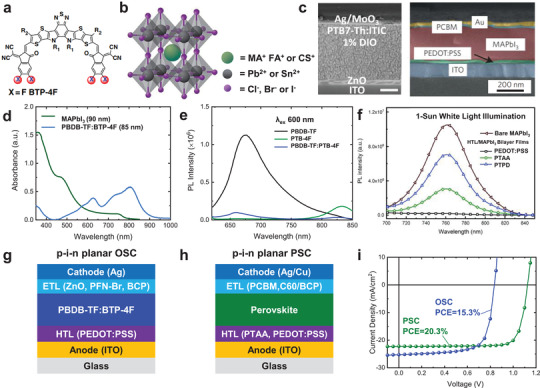
a) Molecular structures of an electron acceptor (BTP‐4F) for organic solar cells.^[^ ^27^ ^]^ b) Perovskite structure where green stands for monovalent cation, gray Pb(II) or Sn(II) cation, and purple is Cl^−^, Br^−^, or I^−^. c) Cross‐sectional scanning electron microscopy (SEM) images of doctor‐bladed organic blend solar cell (SEM bar = 50 nm)^[^ ^28^ ^]^ and inverted p–i–n planar structure perovskite device (SEM bar = 200 nm).^[^ ^29^, ^30^ ^]^ d) Absorbance as a function of wavelength of thin films of MAPbI_3_ (90 nm) and PBDB‐TF:BTP‐4F blend (85 nm).^[^ ^25^, ^31^, ^32^ ^]^ e) PL spectra of PBDB‐TF:BTP‐4F blend films excited on 600 nm for electron transfer from PBDB‐TF.^[^ ^33^ ^]^ f) PL spectra neat MAPbI_3_ film and HTL/MAPbI_3_ bilayer films under 1 sun equivalent white‐light illumination.^[^ ^19^ ^]^ g,h) p–i–n planar device structures for typical PBDB‐TF:BTP‐4F (g)^[^ ^27^ ^]^ and MAPbI_3_ (h) solar cells.^[^ ^34^ ^]^ i) Typical current density–voltage curves for the p–i–n organic (blue line) and MAPbI_3_ (green line) solar cells illustrated in (g) and (h). c) Left: Reproduced with permission.^[^ ^28^ ^]^ Copyright 2018, Wiley‐VCH. c) Right: Reproduced with permission.^[^ ^29^ ^]^ Copyright 2015, Royal Society of Chemistry. e) Reproduced with permission.^[^ ^33^ ^]^ Copyright 2020, Royal Society of Chemistry. f) Reproduced with permission.^[^ ^19^ ^]^ Copyright 2019, Royal Society of Chemistry.

Here, we focus on comparing these processes between OSCs and PSCs, with the hope that this comparison will provide new insights and help the OSC and PSC communities learn and adapt successful strategies from each other.^[^ ^42^ ^]^ While OSCs and PSCs share similar device architectures and often similar charge extraction layers and metal contact (see **Figure** **1**), the intrinsic properties of the light absorbing layers show obvious differences in both systems. These include differences in photoinduced charge generation (exciton separation in OSCs^[^ ^43^ ^]^ vs direct photoexcitation in PSCs^[^ ^44^ ^]^), charge transport (hopping‐like transport in organic semiconductors^[^ ^36^ ^]^ vs band‐like transport in perovskite semiconductors^[^ ^45^ ^]^), the presence of a donor:acceptor bulk heterojunction in most OSCs, and the presence of relatively mobile ions in PSCs.^[^ ^25^ ^]^ Herein, we address how these different intrinsic material properties result in differences in device function and performance, and how knowledge and device models developed for one technology can be successfully applied to the other. We start by focusing in Section 2 on the similarities and differences in device architecture, materials, and operating principles between these two technologies. We move on to discuss charge generation and separation in Section 3, and in particular lessons from ultrafast transient absorption and photoluminescence studies. We address charge transport, trapping, and recombination in Section 4, and the competition between charge extraction and bimolecular recombination in Section 5, focusing in particular on lessons from transient optoelectronic analyses. Finally, we summarize materials and device design guidelines developed from these studies to guide further enhancement in device performance for both OSCs and PSCs in Section 6.

## 2 Device, Material, and Operating Principles

High‐efficiency, solution‐processable photovoltaic devices incorporating either organic blends such as PBDB‐TF:BTP‐4F (also known as PM6:Y6) (where PBDB‐TF = poly[[4,8‐bis[5‐(2‐ethylhexyl)‐4‐fluoro‐2‐thienyl]benzo‐ [1,2‐b:4,5‐b′]dithiophene‐2,6‐diyl]‐2,5‐thiophenediyl‐ [5,7‐bis(2‐ethylhexyl)‐4,8‐dioxo‐4H,8H‐benzo[1,2‐c:4,5‐c′]‐ dithiophene‐1,3‐diyl]‐2,5‐thiophenediyl] and BTP‐4F = 2,2′‐((2Z,2′Z)‐((12,13‐bis(2‐ethylhexyl)‐3,9‐diundecyl‐12,13‐dihydro‐[1,2,5]thiadiazolo[3,4‐e]thieno[2″,3″:4′,5′]thieno[2′,3′:4,5]pyrrolo[3,2‐g]thieno[2′,3′:4,5]thieno[3,2‐b]indole‐2,10‐diyl)bis(methanylylidene))bis(5,6‐difluoro‐3‐oxo‐2,3‐dihydro‐1H‐indene‐2,1‐diylidene))dimalononitrile) or perovskite materials such as MAPbI_3_ can yield short‐circuit photocurrent densities (*J*_SC_) over 20 mA cm^−2^, as shown in Figure 1 (hereafter all discussion of perovskite materials and device properties will refer for simplicity to MAPbI_3_ unless indicated otherwise). However, OSCs with such high photocurrent densities typically exhibit lower open‐circuit voltages (*V*_OC_s) and fill factors (FFs) than comparable PSCs (see, for example, Figure 1).^[^ ^46^ ^]^ This is due to a greater energy loss between the material bandgap and device *V*_OC_ (*E*_LOSS_ = *E*_g_ − *eV*_OC_), which can be as low as 0.34 V for PSC devices, but is typically 0.5–0.7 V for high photocurrent quantum efficiency OSCs.^[^ ^47^, ^48^, ^49^, ^50^ ^]^ Higher *V*_OC_s (≈1.1 V), equivalent to typical efficient PSC devices, have been achieved for OSCs by tuning of donor/acceptor energy levels, but typically at the expense of lower photocurrent densities, resulting either from a higher optical bandgap limiting solar light absorption or a lower internal quantum efficiency. This trade‐off between the high *V*_OC_ and optimal *J*_SC_ is a key performance limitation for OSCs compared to PSCs.

**Table** **1** provides a summary of some of the key materials and device parameters underlying the current–voltage performance of OSCs and PSCs. Perovskite films are significantly more crystalline, with a lower electron–phonon coupling than organic semiconductors. MAPbI_3_ is also more ionic, and exhibits a higher dielectric constant.^[^ ^43^, ^51^, ^64^ ^]^ Excited states in perovskites are more delocalized than in organic materials, due in particular to their lower structural and energetic disorder.^[^ ^65^, ^66^ ^]^ Organic and perovskite semiconductors also differ considerably in terms of exciton binding energy and charge carrier mobility. Strong exciton/charge localization and the low dielectric constant of organic materials enhance electron–hole interactions, as reflected in their strong exciton binding energies (*E*_b_ > 100 meV), whereas electron–hole attraction in perovskite materials is much weaker (*E*_b_ < 25 meV for MAPbI_3_).^[^ ^41^, ^42^, ^43^, ^44^, ^45^, ^46^, ^55^, ^67^, ^68^, ^69^ ^]^ Effective charge carrier mobilities in OSCs (≈10^−5^ to 10^−4^ cm^2^ V^−1^ s^−1^) are usually much lower than those in PSCs (0.1–10 cm^2^ V^−1^ s^−1^),^[^ ^45^, ^70^, ^71^, ^72^ ^]^ with charge transport in organics resulting from hopping transport (small polaron motion), but more band‐like transport in perovskites with a correspondingly small effective mass.^[^ ^73^ ^]^ We will now consider in more detail similarities and differences in the function of these materials, considering first the photophysics of the photoactive layer.

**Table 1:** Comparison of a selection of optoelectronic parameters and functional characteristics of organic and perovskite solar cells

	Organic solar cells^[^ ^51^, ^52^ ^]^	Perovskite solar cells^[^ ^45^, ^53^, ^54^, ^55^, ^56^, ^57^, ^58^ ^]^
Relative dielectric constant (ε_r_)	2–4.0	20–50
Optical bandgap [eV]	1.0–1.6	1.4–1.6
Optimum photoactive layer thickness [nm]	≈100	200–700
Effective carrier mobility^a)^ [cm^2^ V^−1^ s^−1^]	10^−5^ to 10^−4^	0.1–10
Tail state characteristic energy^b)^ [meV]	27–100	40–100
Charge carrier density at open circuit in 1 sun [cm^−3^]	10^16^ to 10^17^	10^15^ to 10^16^
Charge carrier lifetime at open circuit [s]	10^−5^ to 10^−6^	≈10^−6^
Charge carrier diffusion length at open circuit [nm]	≈20	>500
Mechanism of charge generation	Exciton separation at donor/acceptor interface	Direct photoexcitation
Ion migration	No	Yes
Field in the bulk at short circuit	Yes	No
Charge carrier transport at short circuit	Drift	Diffusion
Requirement of contacts for efficient photocurrent collection	Work function difference to generate built‐in potential	Carrier selective contacts

^a)^ The effective mobility values of organic solar cells and perovskite solar cells list here are measured by transient optoelectrical method charge extraction (CE)^[^ ^59^, ^60^ ^]^

^b)^ Tail state characteristic energies determined from transient optoelectrical methods (TPV/CE), as discussed here; for comparison, optical Urbach energies, measured by Fourier transform photocurrent spectroscopy, for OSCs are typically 27–63 meV and for PSCs are 14–16 meV.^[^ ^61^, ^62^, ^63^ ^]^

## 3 Charge Generation and Separation

Charge generation in both OSCs and PSCs is driven by light absorption of photoactive materials. In PSCs, light absorption directly generates separated charge carriers. For OSCs, charge generation requires the separation of exciton and/or charge transfer states, and is therefore more complex, and more critical to device efficiency. In this section, we focus on steady‐state and transient spectroscopic studies of charge generation and separation in OSCs and PSCs.

### 3.1 Material Absorbance and Photoluminescence

Both organic blends and perovskites are stronger light absorbers than silicon, the most widely employed photovoltaic material, thus both OSCs and PSCs can reach similar photocurrent quantum efficiencies as Si‐based solar cells but with thinner active layers. Organic donor:acceptor blends typically exhibit stronger visible/near‐IR light absorption than perovskite absorbers for matched film thicknesses, as illustrated for MAPbI_3_ and PBDB‐TF:BTP‐4F films in Figure 1d (MAPbI_3_ exhibits stronger blue/UV absorption, less useful for solar light harvesting). However, the performance of most bulk heterojunction OSCs drops off for active layer thicknesses greater than 100 nm, attributed to recombination losses during charge extraction.^[^ ^66^, ^74^, ^75^ ^]^ By contrast, PSCs exhibit efficient charge extraction for active layer thicknesses of >0.5 µm,^[^ ^76^, ^77^ ^]^ attributed to their high carrier mobilities as we will discuss further below.^[^ ^58^, ^78^ ^]^ This enables efficient solar light absorption across the visible and near‐IR in both technologies. As such, differences in quantum efficiencies for photocurrent generation between OSCs and PSCs do not typically result from differences in light absorption, but rather from the differences in the underlying photophysics and charge carrier dynamics, the primary topic of this perspective.

Photoluminescence (PL) quenching is one of the simplest assays to measure the yield of charge transfer between electron donor and electron acceptor in OSCs or from perovskite bulk to charge transport layers in PSCs. For organic semiconductor blends, PL emission comes mostly from Frenkel excitons in pure domains; on the other hand, for perovskite semiconductors, PL emission originates primarily from radiative bimolecular recombination of unbound charges. Optimal PL emission quantum yields of neat organic and perovskite films are of similar magnitudes, but show great variability depending upon material selection (for organics) and processing (for perovskites). PL emission from perovskites is narrower, indicative of less energetic disorder of the radiative states compared to those in organic materials. We can link this sharper emission to organic materials being softer, with more phonon modes and more localized excited states, enhancing electron–phonon coupling and leading to PL broadening.^[^ ^79^, ^80^, ^81^, ^82^ ^]^ Similarly, with notable exceptions,^[^ ^83^ ^]^ perovskite materials show smaller optical Urbach tails to their absorption onsets than organic semiconductors.^[^ ^84^, ^85^, ^86^, ^87^ ^]^ Organic donor:acceptor blends show strongly quenched emission relative to neat materials, attributed to exciton dissociation and subsequent nonradiative charge recombination at the donor/acceptor interface; this PL quenching is widely used as an assay of efficient exciton separation in organic blends.^[^ ^88^, ^89^, ^90^, ^91^ ^]^ By contrast, in neat perovskite films, PL emission comes from the radiative recombination of free charge carriers, with the main competing pathway being charge localization into nonradiative trap states.^[^ ^41^, ^92^ ^]^ Such nonradiative charge trapping is most dominant at low light fluxes, but becomes at least partially suppressed at high light intensities (e.g., 1 sun), attributed to trap filling.^[^ ^93^, ^94^, ^95^, ^96^ ^]^ In perovskite/charge transport layer stacks, the presence of the charge transport layers can also result in PL quenching, with the magnitude of this quenching being dependent on light irradiation intensity.^[^ ^97^, ^98^ ^]^ This PL quenching can be used as an assay of the efficiency of charge extraction in complete PSCs.^[^ ^98^, ^99^, ^100^, ^101^, ^102^ ^]^ For both efficient OSCs and PSCs, absolute PL quantum yields are relatively low, even at open circuit (typically <<10%),^[^ ^103^ ^]^ indicating that for both devices radiative recombination (of either excitons or charges) is not the primary limit to practical device efficiency (although they do impose limits on theoretically achievable efficiencies). PSCs typically exhibit higher electroluminescence (EL) yields than OSCs,^[^ ^104^, ^105^ ^]^ consistent with the greater dominance of radiative rather than nonradiative charge recombination in these devices. The greater dominance of nonradiative charge recombination in OSCs is likely to be a fundamental limit to the efficiencies achievable for this technology. In organic materials, singlet exciton decay, primarily by nonradiative recombination to ground or triplet states, limits exciton diffusion lengths to typically <10 nm, less than the optical absorbance depth, requiring the use of bulk heterojunction to enable exciton separation. In addition, lower‐bandgap organic semiconductors tend to have enhanced nonradiative exciton decay due to the energy gap law, likely to further limit exciton lifetimes and diffusion lengths,^[^ ^106^, ^107^ ^]^ although we note clear exceptions to this trend.^[^ ^38^ ^]^

### 3.2 Ultrafast Charge Carrier Dynamics

The most widely employed experimental technique to observe exciton and charge generation in organic and perovskite layers is ultrafast transient absorption spectroscopy. This is a pump–probe technique used to monitor transient optical absorption/transmission changes associated with photoexcited states, typically observed on femtosecond to nanosecond timescales. Femtosecond laser pulses are used as an excitation source, while typically white light probe pulse determines the change in the absorbance of the film or device induced by the laser excitation. As such, the magnitude of the transient absorption (ΔOD = change in the optical density) can be employed as a direct, at least semiquantitative, assay of the yield and kinetics of exciton and charge generation. Transient photoluminescence studies are also widely employed to probe the decay of emissive photoexcited states (singlet excitons in OSCs, charge pairs in PSCs). Time correlated single photon counting (TCSPC) is the most widely used transient photoluminescence technique, employing lower excitation densities than most other ultrafast techniques, but exhibiting a slower time resolution (typically tens to hundreds of picoseconds).

**Figure 2:**
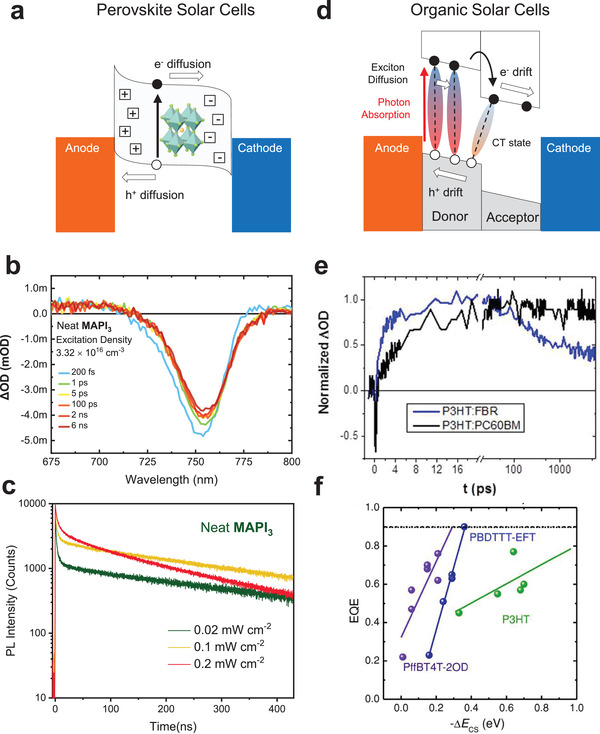
a) Illustration of the band diagram and main processes in perovskite solar cells. b) Transient absorption spectra of a MAPbI_3_ film for time delays up to 6 ns acquired at an excitation density of 3.3 × 10^16^ cm^−3^.^[^ ^98^ ^]^ c) Time‐correlated single photon counting (TCSPC) decay dynamics of neat MAPbI_3_ films measured under 0.02–0.2 mW cm^−2^ excitation densities (0.02–0.2% of 1 sun).^[^ ^98^ ^]^ d) Illustration of band diagram and main processes in organic solar cells. e) Rise and decay of P3HT:FBR and P3HT:PC_60_BM blend polaron signals measured using transient absorption spectroscopy under 4 µJ cm^−2^ excitation density on picosecond timescale, excited at 600 nm and probed at 725 nm.^[^ ^108^ ^]^ f) External quantum efficiency (EQE) as a function of energy offset −Δ*E*_CS_ for OSC devices employing PffBT4T‐2OD (purple), PBDTTT‐EFT (blue), and P3HT (green) as an electron donor and various nonfullerene electron acceptors. The solid lines have been obtained by using a linear fit analysis and the dashed line in the panel at EQE = 0.9 corresponds to near unity internal charge generation efficiency.^[^ ^11^ ^]^ b,c) Reproduced with permission.^[^ ^98^ ^]^ Copyright 2018, Wiley‐VCH. e) Reproduced with permission.^[^ ^108^ ^]^ Copyright 2014, American Chemical Society. f) Reproduced with permission.^[^ ^11^ ^]^ Copyright 2018, Wiley‐VCH.

For both OSCs and PSCs, the photovoltaic function is based upon the photogeneration of separated charges within their light absorber layers (**Figure** **2**). With some notable exceptions, such as 2D structures and bromide containing perovskites,^[^ ^69^, ^109^ ^]^ photoexcitation of most organohalide perovskite films directly generates free electrons and holes (Figure 2a). This can be attributed to the low exciton binding energies in such materials resulting from their high dielectric constant and highly crystalline structure.^[^ ^55^, ^110^, ^111^, ^112^ ^]^ As such, the ultrafast (femtosecond–picosecond) charge carrier kinetics of perovskite films are often much less important in determining device performance than those of OSCs. Typical transient absorption spectra as a function of time delay for a MAPbI_3_ film are illustrated in Figure 2b for an excitation density of 3 × 10^16^ cm^−3^ (similar to the charge density under 1 sun conditions in a device at open circuit).^[^ ^98^ ^]^ A negative signal is observed at 760 nm (approximately MAPbI_3_'s absorption onset) assigned to the ground‐state bleach signal from photogenerated free carriers. A small initial (<1 ps) signal decay is apparent, assigned to hot carrier cooling to the band edges.^[^ ^113^ ^]^ After this decay, the transient signal is essentially invariant from 1 ps to 6 ns, indicating that, under these ≈1 sun charge density conditions, there is negligible recombination of these photogenerated free charge carriers. At higher excitation densities, these charge carriers are observed to decay more quickly, (e.g., as an approx. 1 ns decay at 3 × 10^18^ cm^−3^),^[^ ^96^ ^]^ assigned carrier‐density‐dependent bimolecular recombination.^[^ ^98^, ^114^ ^]^ At lower excitation densities, most typically probed by TCSPC transient PL measurements, a monomolecular ≈1 ns decay phase can be observed, attributed to charge localization into nonradiative trap states, as shown in Figure 2c. This fast phase assigned to charge trapping is suppressed by trap filling at higher excitation densities (Figure 2c),^[^ ^93^, ^94^ ^]^ and is also suppressed in more crystalline films, attributed to lower trap densities due to film processing.^[^ ^113^, ^115^ ^]^ The TCSPC data in Figure 2c also exhibit a slow (a few hundreds of nanoseconds, intensity‐dependent) PL decay phase assigned to bimolecular recombination. The lifetime of this decay phase is also dependent on excitation density and film processing, and can be as long as 4 µs for highly crystalline films.^[^ ^116^ ^]^ It accelerates at higher excitation densities (see Figure 2c), consistent with its assignment to bimolecular recombination. The presence of charge transport layers can result in a much faster PL decay, assigned to charge transfer to these transport layers. The efficiency of this transfer (extraction) process is strongly dependent on excitation density, being limited at low excitation densities by charge trapping, and at high excitation densities by bimolecular recombination.^[^ ^98^ ^]^

In contrast to PSCs, ultrafast charge carrier kinetics are critical to the performance of OSCs. As illustrated in Figure 2d, the low dielectric constant of organic semiconductors results in photoexcitation generating Frenkel excitons.^[^ ^51^, ^55^, ^117^, ^118^ ^]^ In neat organic semiconductor films, these excitons exhibit lifetimes ranging from ≈20 ps to ≈1 ns and exciton diffusion lengths from ≈3 to 20 nm.^[^ ^106^, ^119^ ^]^ These excitons are primarily separated by charge transfer at organic donor/acceptor interfaces, driven by energetic offsets between donor and acceptor electronic energy levels.^[^ ^105^, ^120^ ^]^ A bulk heterojunction (BHJ) blend morphology is typically employed to facilitate photogenerated excitons accessing this interface. While this BHJ is often illustrated as a bicontinuous interpenetrating network of nanoscale donor and acceptor domains, in practice most organic BHJ blends comprise a high proportion of molecularly mixed domains alongside pure (or purer) domains of donor and/or acceptor.^[^ ^121^, ^122^ ^]^ Following exciton separation, charges may either dissociate into free charges, or remain at donor/acceptor interfaces as electron–hole pairs. The presence of interfacial electron–hole pairs can be most readily observed by the observation of charge transfer absorption and emission redshifted from the absorption and emission of the neat materials, as such these interfacial states are often referred to as charge transfer (CT) states. Studies have reported both Coulombically bound CT states, which subsequently undergo geminate (monomolecular) recombination, and relatively unbound CT states, which dissociate into free charges to generate photocurrent.^[^ ^123^, ^124^, ^125^, ^126^ ^]^ Typical transient absorption data illustrating these processes are shown in Figure 2e for a blend of the polymer donor P3HT with molecular acceptors FBR or PC_60_BM.^[^ ^108^ ^]^ For P3HT:FBR, the rise kinetics (assigned to polaron formation from P3HT excitons) are significantly faster compared to P3HT:PC_60_BM, and dominated by an instrument response limited rise, consistent with the more intermixed P3HT:FBR blend morphology reducing exciton diffusion limitations. However, the P3HT:FBR blend also exhibits a 200 ps decay phase not observed for P3HT:PC_60_BM, assigned to the geminate recombination of CT states resulting from the smaller LUMO–LUMO energy offset (LUMO = lowest unoccupied molecular orbital) and/or more intermixed morphology of this blend. This geminate recombination reduces the photocurrent density observed for P3HT:FBR devices.

For many organic donor:acceptor blends, the internal quantum efficiency of photocurrent generation is limited by the efficiencies of exciton and/or CT state dissociation. Field‐dependent CT state dissociation can also result in limitations to the device fill factor.^[^ ^127^, ^128^ ^]^ Film nanomorphology has a critical role in minimizing these losses, with a more intermixed morphology reducing exciton losses during their diffusion to donor/acceptor interfaces. However, a more intermixed morphology also results in greater recombination losses (see Figure 2e), with CT states generated within intermixed domains, rather than at donor/acceptor interfaces with pure domains, being more likely to undergo CT state (geminate) recombination rather than dissociate into free charges.^[^ ^88^ ^]^ Charge separation at donor/acceptor interfaces is driven, at least in part, by HOMO–HOMO and LUMO–LUMO orbital energy offsets (Δ*E*) (HOMO = highest occupied molecular orbital). This energy offset requirement has often been considered a fundamental limit to OSC efficiency which is not present in perovskite solar cells. A larger energy offset is often correlated with suppressed CT state recombination losses, but also reduces the blend electronic bandgap (IP_donor_ − EA_acceptor_) limiting device *V*_OC_ (IP = ionization potential; EA = electron affinity) As such, a trade‐off between the *V*_OC_ and *J*_SC_ is often observed in OSCs; a small energy offset increases *V*_OC_ but limits the efficiency of charge separation, and vice versa for a large energy offset. However, it has also been observed that the magnitude of the energy offset required for efficient photocurrent generation varies between classes of donor or acceptor materials (Figure 2f), being, for example, larger for blends employing polythiophene donor polymers than for those employing PffBT4T‐2OD and PBDTTT‐EFT (PffBT4T = poly[(5,6‐difluoro‐2,1,3‐benzothiadiazol‐4,7‐diyl)‐*alt*‐(3,3″′‐di(2‐alkyl)‐2,2′,5′,2″,5″,2″′‐quaterthiophen‐5,5″′‐diyl)]; PBDTTT‐EFT = poly[4,8‐bis(5‐(2‐ethylhexyl)thiophen‐2‐yl)benzo[1,2‐b;4,5‐b']dithiophene‐2,6‐diyl‐*alt*‐(4‐(2‐ethylhexyl)‐3‐fluorothieno[3,4‐b]thiophene‐)‐2‐carboxylate‐2‐6‐diyl)]).^[^ ^11^, ^36^ ^]^ Indeed, there are now examples of donor:acceptor blends, such as PBDB‐TF:BTP‐4F (Figure 1) which have been suggested to achieve efficient photocurrent generation with near zero energy differences between their exciton energies (i.e., their optical bandgap) and their donor/acceptor band edges (i.e., IP_donor_ − EA_acceptor_),^[^ ^33^, ^129^ ^]^ (see however ref. ^[^ ^130^ ^]^ for an alternative estimate of the energy offset in this system). In such low energetic driving force blends, it is likely that, as for perovskite films, charge separation is stabilized by the low density of photogenerated charges reducing the likelihood of subsequent bimolecular recombination events. In other words, it appears likely that in both perovskite and low energy offset OSCs, charge separation is primarily stabilized by entropy increase resulting from generating two spatially uncorrelated charges.^[^ ^131^, ^132^, ^133^ ^]^ This entropic stabilization is equivalent to the difference between the quasi‐Fermi level splitting (QFLS), (corresponding to the free energy stored) and the electronic bandgap (corresponding to the enthalpy stored, see our previous work^[^ ^35^ ^]^ for further discussion of this point).

The formation of excitons in OSCs, rather than the direct free charge generation observed in PSCs, impacts strongly on the ultrafast kinetic requirements for efficient function, as discussed above. However, in terms of thermodynamics, the presence of excitons, and an exciton binding energy, does not appear to be a fundamental limit to OSC efficiency relative to PSCs. Overcoming this exciton binding energy (*E*_b_) requires the presence of an energy offset (Δ*E*) between donor/acceptor HOMO and LUMO levels to drive separation, reducing the electronic bandgap of the blend, and thus reducing device *V*_OC_. However, the exciton binding energy also reduces the optical bandgap of each material, enhancing light absorption and thus photocurrent density. In the limit of efficient free charge generation with *E*_b_ ∼ Δ*E*, these two factors should approximately cancel, with no net impact on device performance. Progress toward achieving such efficient charge generation with *E*_b_ ∼ Δ*E* (i.e., a near zero enthalpic driving force for exciton separation) has been suggested to be a key factor behind recent advances in the efficiency of OSCs with nonfullerene acceptors,^[^ ^33^, ^38^, ^129^, ^134^ ^]^ although this point has been disputed.^[^ ^135^ ^]^ It is likely that high local carrier mobilities and low electron–phonon coupling aid efficient charge generation with low energetic offsets,^[^ ^35^ ^]^ although this has not been well established. Long exciton and CT state lifetimes can also aid efficient charge separation, reducing the kinetic competition between separation and decay to the ground.^[^ ^38^, ^136^, ^137^ ^]^

## 4 Charge Recombination, Trapping, and Transport

After the generation of free charge carriers, for both OSCs and PSCs, the quantum efficiency of charge collection is limited by recombination losses during charge transport and extraction. Charge trapping can also reduce the yield of charge collection, as well as lowering the energy of accumulated charges. Shallow trap states (tail states) result in reversible charge trapping, while deep trap states result in effectively irreversible trapping and recombination. Here, we use the term bimolecular recombination to refer to the recombination of photogenerated charge carriers accumulated in either band states or shallow tail states. In this section, we primarily focus on the use of transient optoelectronic methods to assay these charge accumulation, recombination, trapping, and transport processes in both OSCs and PSCs. We discuss the strengths and limitations of these methods compared to other approaches, including, for example, consideration of surface recombination and charge accumulation on device contacts, and the lessons which can be derived from such studies in terms of OSC and PSC device function and efficiency.

Free charge carriers in organic semiconductors are small polarons, associated with a significant lattice relaxation around each charge. In perovskite materials, such polaronic effects are thought to be less severe, with free charge carriers being relatively delocalized, and associated with smaller lattice distortions.^[^ ^138^, ^139^ ^]^ It appears likely that the greater energetic relaxation associated with polaron formation and charge trapping in organic materials is one of the key underlying reasons behind the larger energy loss (*E*_g_ − *qV*_OC_) observed for OSCs compared to PSCs. We also note that the energetic relaxation associated with polaron formation results in an energetic stabilization of charges relative to excitons, and therefore may aid charge separation in organic blends. In addition, polaron formation is a key factor underlying the lower carrier mobilities of organic semiconductors compared to perovskites. Carrier mobility is critical in determining the kinetics of charge transport to the device contacts for collection by the external circuit. For both devices, the efficiency of this charge transport is limited by competition between charge extraction and recombination processes.^[^ ^140^, ^141^ ^]^ Before considering these processes in more detail, we consider these recombination processes at open circuit, where charge extraction is absent.^[^ ^33^ ^]^

### 4.1 Open‐Circuit Voltage

The open‐circuit voltage (*V*_OC_) is a key determinant of solar cell device performance. It is ultimately limited by the electronic bandgap of the device, and thus determined primarily by material energetics.^[^ ^142^, ^143^, ^144^ ^]^ However, how closely *V*_OC_ (or more specifically the quasi‐Fermi level splitting in the bulk) can approach this electronic gap also depends upon charge carrier recombination kinetics. As the electron and hole Fermi levels approach the band edges, the charge density in the device increases exponentially, resulting in a rapid acceleration of bimolecular electron/hole recombination, which limits device *V*_OC_.^[^ ^144^ ^]^ Most models to describe *V*_OC_ explicitly include the impact of charge recombination; however, experimental approaches to directly probe these recombination kinetics remain controversial, particularly for PSCs. In this section, we will show how device *V*_OC_ can be accurately reconstructed for a wide range of OSCs and PSCs via transient optoelectronic measurements of charge carrier lifetimes and charge carrier densities. We will also discuss limits to the validity of such transient optoelectronic measurements, and in particular under what conditions they directly probe the QFLS in the photoactive layer of OSC and PSC devices.

**Figure 3:**
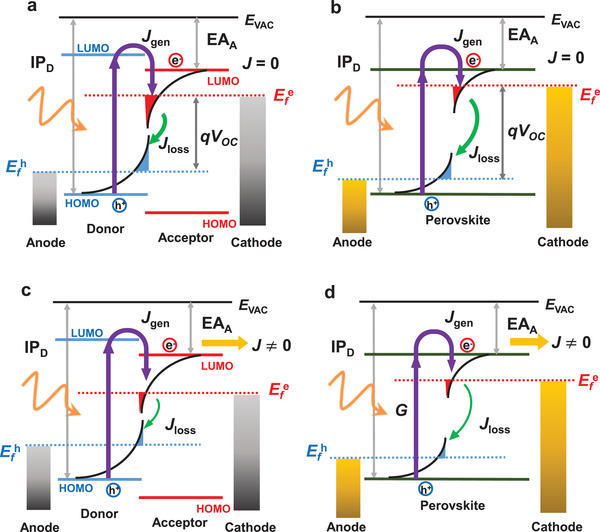
A simplified schematic of the balance between charge carrier photogeneration (illustrated as *J*_gen_) and charge recombination (illustrated as a recombination loss current *J*_loss_) and external current (*J*) under light illumination in OSCS (left) and PSCs (right). a) Open‐circuit condition (*J* = 0) for an OSC, with the contact potential difference (*V*_OC_) assumed to track the quasi‐Fermi level splitting (QFLS) in the bulk (i.e., neglecting QFLS losses at the device contacts). The figure includes band‐edge tail states (shallow trap states) extending into the effective bandgap, with shading indicating filled tail states. b) Open‐circuit condition for an equivalent PSC. c,d) Illustrations under charge extraction (e.g., at the maximum power point (MPP)) for an OSC (c) and for a PSC (d).

At *V*_OC_, shown in a simple model in **Figure** **3** (label (a) for OSC and label (b) for PSC), and therefore in the absence of any external charge extraction, the rate of generation of charges (refer to as a generation current density *J*_gen_) under continuous irradiation must be equal and opposite to the rate of charge recombination (referred to as a recombination loss flux *J*_loss_). The recombination rate balances with the generation rate, thus a light‐induced quasi‐Fermi splitting is reached at a steady state. If we assume that *J*_gen_ is independent of device voltage and that *J*_loss_ is negligible at short circuit (often valid due to the lower charge density at short circuit), then in this simple model, *J*_gen_ can be approximated to *J*_SC_ and we can conclude that at *V*_OC_

(1) $$J\left(V\right)=J_{\text{gen}}-J_{\text{loss}}\left(V\right)\approx J_{\text{SC}}-\left(edn/\tau_{n}\right)=0$$

where *n* is the additional charge carrier density generated by photoexcitation and *τ_n_* is the corresponding charge carrier lifetime. *n* can be measured by a full signal measurement such as charge extraction (CE) or by the integration of a small perturbation measurement such as differential charging (DC). Both CE and DC are usually employed to determine the open‐circuit charge density, although CE measurements can also be employed across the *J*–*V* curve (see Sections 4.2 and 5.1 below). *τ_n_* is often measured by transient photovoltage (TPV) decays, typically employing small laser‐induced perturbations at open circuit as a function of background light intensities.^[^ ^145^, ^146^, ^147^, ^148^, ^149^, ^150^, ^151^, ^152^ ^]^ As discussed below, *n* can be determined in cm^−2^ or cm^−3^, with the latter case only possible when these charge carriers are primarily located in a single component of the device (i.e., the photoactive layer) and distributed spatially homogeneously. Impedance analyses can also be employed to measure both parameters.^[^ ^153^, ^154^ ^]^ *n* is typically observed to increase exponentially with *V*_OC_, *n* = *n*_0_exp(*γV*_OC_), and *τ_n_* to show a power law dependence on *n* (τ*_n_* = τ_0_ *n* ^−^ *^λ^*) where *n*_0_, τ_0_, γ, and λ are fitting constants. In this case, the recombination loss current can be rewritten as 
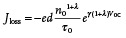
. At open circuit, *J*_loss_ = *J*_gen_ ≅ *J*_SC_, thus

(2)

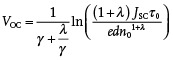




**Figure 4:**
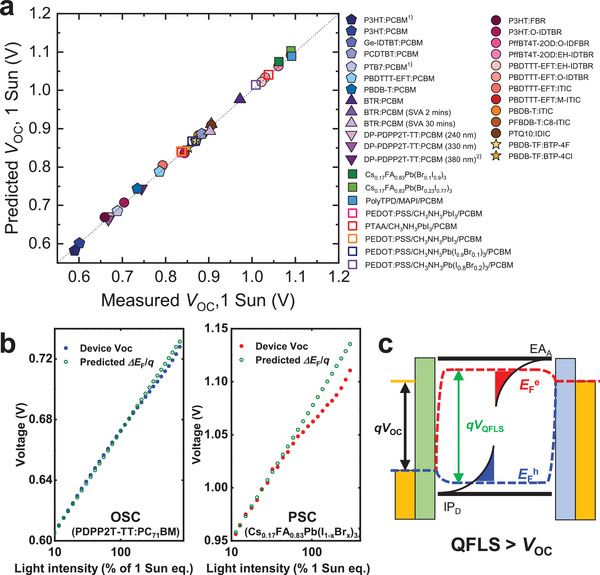
a) The calculated *V*_OC_ versus directly measured *V*_OC_ of a series of organic solar cells and perovskite solar cells. Reconstructed *V*_OC_ determined from Equation (2). The open squares refer to perovskite devices with built‐in potential *V*_BI_ smaller than the QFLS in the bulk. b) The light‐intensity‐dependent predicted QFLS obtained from Equation (3) versus directly measured *V*_OC_ in PDPP2T‐TT:PC_71_BM and Cs_0.17_FA_0.83_Pb(I_1−_ *_x_* Br*_x_*)_3_ devices. c) The schematic illustration of Fermi level bending at the interface when the QFLS is greater than the open‐circuit voltage tracked on the contacts. The *J*–*V* performances of the devices shown here were published previously, their fabrication details can be found in the corresponding references.^[^ ^5^, ^19^, ^33^, ^66^, ^78^, ^148^, ^155^, ^156^, ^157^, ^158^ ^]^ For OSCs with superscript 1), PCBM refers to PC_61_BM, for other OSCs, PCBM refers to PC_71_BM.

It follows from Equation (2) above that experimental measurement of *J*_SC_, *n*, and *τ_n_* as a function of light intensity allows calculation of *V*_OC_ from transient optoelectronic data and *J*_SC_.^[^ ^61^, ^146^ ^]^ Comparison of this independently calculated *V*_OC_ versus the directly measured *V*_OC_ allows the determination of whether this simple model, and its assumptions, are valid for a particular device studied. **Figure** **4**a shows a plot of calculated *V*_OC_ versus the directly measured *V*_OC_ for a wide range of both OSC and PSC devices. An excellent agreement is observed, with calculated *V*_OC_ agreeing with the measured *V*_OC_ within ±5 mV for both classes of devices, indicating that these devices, these analysis tools, and this simple model for *V*_OC_, work remarkably well.

The model detailed herein for *V*_OC_ is significantly more accurate at calculating the measured *V*_OC_ than *V*_OC_ analyses based upon energetic measurements alone (for example, correlations between *V*_OC_ and electronic bandgap or CT state energy of the donor:acceptor blend for OSCs), as the model accounts for differences not only in material energetics (*n*(*V*)) but also charge carrier recombination kinetics (τ(*n*)). An alternative way to evaluate the voltage loss associated with recombination contributions is through analyzing the EL quantum efficiency,^[^ ^159^, ^160^ ^]^ which also improves the accuracy of determining device *V*_OC_ compared to solely energetic analyses.^[^ ^107^, ^161^ ^]^ For many devices, the model for *V*_OC_ detailed above is also able to reproduce the dependence of *V*_OC_ upon light intensity (see, for example, Figure 4b), except at particularly high or low light fluxes, as discussed further below. This model is useful at discriminating between differences in *V*_OC_ due to differences in either material energetics or recombination kinetics. For example, the relatively low *V*_OC_ measured for DP‐PDPP2T‐TT:PC_71_BM devices (DP‐PDPP2T‐TT = poly[2,5‐bis(2‐decylnonadecyl)pyrrolo[3,4‐c]pyrrole‐1,4‐(2H,5H)‐dione‐(E)‐1,2‐di(2,2′‐bithiophen‐5yl)ethene]) results from its faster recombination kinetics compared to most OSCs in this plot (see also Figure 4b below); these faster kinetics lowers *V*_OC_ by about 100 meV compared to other blends with similar energetics.^[^ ^59^ ^]^ In polythiophene‐based OSCs, a tenfold reduction in charge carrier lifetime has found to result in a 100 mV loss of *V*_OC_.^[^ ^145^ ^]^ By contrast, the relatively high *V*_OC_ obtained in OSC with NFAs such as IDTBR result from a wider electronic bandgap (lower *n*(*V*)) rather than slower recombination. Similarly, for perovskite devices, the lower *V*_OC_s observed with poly(3,4‐ethylenedioxythiophene):poly(styrenesulfonate) (PEDOT:PSS) interlayers result from more severe surface recombination losses in these devices compared to, for example, poly[bis(4‐phenyl)(2,5,6‐trimethylphenyl)amine (PTAA) interlayers,^[^ ^19^ ^]^ as we discuss further below. As such, this model and analysis is able to provide key insights into the factors that determine *V*_OC_ in both organic and perovskite solar cells.

It should be noted that the analyses and model detailed above do not correctly calculate the measured *V*_OC_ for all OSCs and PSCs. For example, in OSCs where charge generation is field‐dependent, *J*_SC_ cannot be used as a proxy for *J*_gen_ at open circuit, requiring a separate measurement of *J*_gen_(*V*). For OSCs with low charge carrier mobilities, the charge equilibrium time between the bulk and contact can be slower than the recombination time, this can limit the use of TPV decay to assess the recombination constant in some devices.^[^ ^162^ ^]^ In perovskite devices, the correct calculation of *V*_OC_ is complicated by ion motion within the perovskite layer. In PSCs, a valid charge density (*n*) is normally only obtained by differential charging, with charge extraction giving anomalous charge densities, most likely resulting from ion motion in the perovskite during the CE measurement.^[^ ^148^ ^]^ Similarly, TPV and impedance analyses of charge carrier lifetimes can also be complicated by ion motion on the timescale of these measurements.^[^ ^163^, ^164^, ^165^ ^]^ Normally, such optoelectronic analyses work best on PSCs which exhibit minimal hysteresis, such as devices with organic charge collection layers.^[^ ^78^, ^166^ ^]^ As such, confirmation of a correct *V*_OC_ reconstruction is an essential prerequisite for using these optoelectronic measurements as assays of charge carrier lifetimes and densities in both PSCs and OSCs.

**Figure 5:**
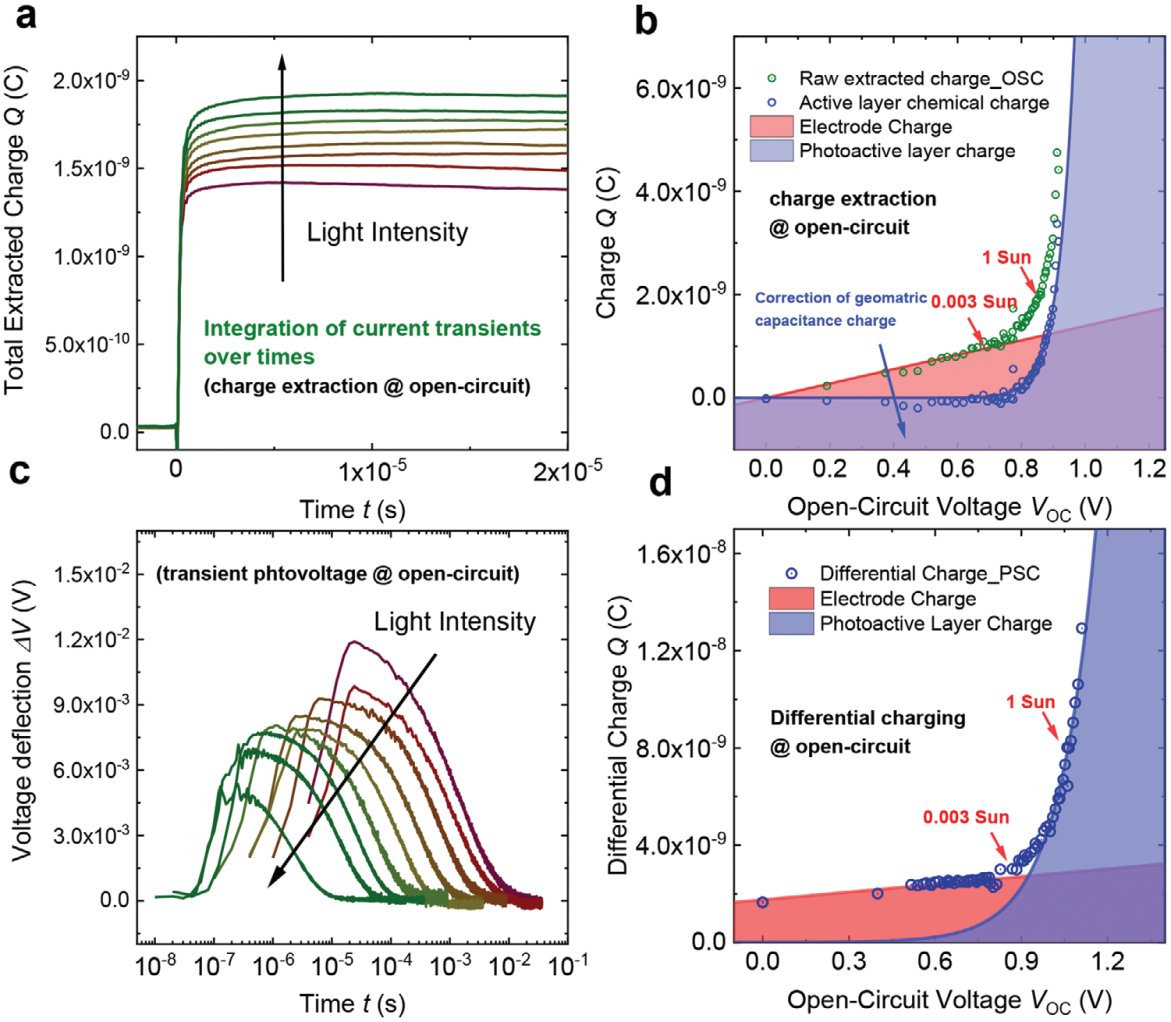
a) Integrated charge extraction transients measured over a range of light intensities for an organic solar cell (PM6:Y6). b) Extracted charge measured from such charge extraction data (extracted from light‐induced open‐circuit condition) as a function of OSC *V*_OC_ determined either without (green circles) or after (blue circles) subtraction of the calculated geometric contact capacitance corrected charge (blue circles). c) Photovoltage transients induced by a small laser pulse perturbation (<7 ns) at open circuit as a function of induced voltage deflections (linear) as a function of time (logarithm) over a range of background light intensities for a perovskite solar cell (CsFAPbI). d) Differential charge determined from these photovoltage transients and a corresponding photocurrent transient as a function of PSC *V*_OC_. For both OSC and PSC devices, data collected over a range of background light intensities from 0 to 10 sun equivalents. The linear increase in charge at low light intensities/*V*_OC_s is assigned to charge accumulation on the device contacts and the sharp (exponential) increase in charge observed at high light intensities/*V*_OC_s is assigned to charge accumulation in tail/band states of the photoactive layer of the device.

The analysis and *V*_OC_ reconstruction detailed above is independent of the physical location of the charges in the device—for example, working both for devices where charge density at *V*_OC_ is primarily located either in the photoactive layer or on the device contacts. The physical location of charge carriers can be most readily identified from plots of *n* (calculated as cm^−2^) versus *V*_OC_. **Figure** **5** illustrates charge extraction and differential charging analyses of photoinduced charge accumulation in an OSC (Figure 5a,b) and a PSC (Figure 5c,d). Charge located on device contacts (or doped interlayers) typically increases linearly with *V*_OC_, corresponding to a constant geometric capacitance. Confirmation of this assignment can be made by comparison with *C*_geo_ = *εA*/*d* (where ε is the dielectric constant of the photoactive layer, *A* the device area, and *d* is the photoactive layer thickness). Charge in the photoactive layer typically increases exponentially with *V*_OC_, as the electron and hole Fermi levels approach the band edges, often referred to as the chemical capacitance *C*_chem_ (it should be noted that undoped interlayers can also generate exponentially increasing capacitances, complicating data interpretation). At low light intensities, the geometric capacitance tends to dominate, while at higher light intensities, the exponential increase in active layer charge density results in the active layer chemical capacitance dominating.^[^ ^60^, ^148^ ^]^ When most charge carriers reside on the device contacts, *τ_n_* is often referred to as a discharge time constant.^[^ ^167^, ^168^ ^]^ In this limit, if the discharge time is primarily via bimolecular recombination through the bulk, measurement of total capacitance and effective charge carrier lifetime can still allow calculation of the bimolecular recombination rate constant.^[^ ^168^, ^169^ ^]^ In most efficient OSC and (more arguably) PSC devices under 1 sun irradiation at open circuit, most charge resides in the photoactive layer, *C*_chem_ >> *C*_geo_.^[^ ^144^, ^148^ ^]^ In this limit, the charge carrier lifetimes determined from, for example, TPV measurements can correspond to measurements of the bimolecular recombination in the active layer of the device, as discussed further below.

For both OSCs and PSCs, charge photogeneration in the photoactive layers leads to a QFLS (Δ*E*_F_ = *E*_F_^n^ − *E*_F_^p^) in the bulk. *V*_OC_ is measured by the potential difference between two metal contacts of the device. For a photovoltaic device with ideal contacts (i.e., not inducing contact energetics/recombination losses), and assuming that charge equilibrium between the bulk and the contacts is faster than charge recombination losses (apparent as transient photocurrent (TPC) decay times being faster than TPV decay times), the contact potential difference (*V*_OC_) will direct track the photogenerated QFLS in the bulk.^[^ ^170^ ^]^ In this limit, it is possible to calculate the QFLS in the device by measurement of the charge carrier lifetimes τ and densities *n* as a function of light intensity at open circuit, and then applying the equation

(3) $$J_{\text{loss}}=edR=ed\frac{dn}{dt}=ed\frac{n_{0}\exp\left(\gamma V_{\text{OC}}\right)}{\tau_{0}\exp\left(-\lambda \gamma V_{\text{OC}}\right)}=edR_{0}\exp\left(\frac{QFLS}{n_{\text{id}}kT}\right)$$

where *n*_0_, γ, τ_0_, and λ are experimentally derived constants from TPV/CE, *R*_0_ is the equilibrium recombination rate, *n*_id_ is the ideality factor, and *kT* is the thermal energy.^[^ ^144^ ^]^ We note that QFLS in PSC devices can also be assayed by photoluminescence measurements, as discussed in the section below.

Both OSCs and PSCs can suffer from surface recombination losses at the active layer/contact interface. In the presence of such surface recombination, the equivalence of *V*_OC_ and QFLS breaks down. Such surface recombination is particularly an issue for PSCs.^[^ ^18^, ^171^, ^172^, ^173^ ^]^ For example, in PSCs, *V*_OC_ has been observed to be lower in devices employing PEDOT:PSS as a hole‐transport layer (HTL) rather than PTAA or poly(*N*,*N*′‐bis‐4‐butylphenyl‐*N*,*N*′‐bisphenyl)benzidine (PTPD) assigned, as we discuss further below, to a Fermi level drop at the contact due to greater surface recombination.^[^ ^19^, ^170^ ^]^ We note that the *V*_OC_ reconstruction methodology detailed above can work even in the presence of such surface recombination losses (see, for example, the PSC data points in Figure 4a, where the *V*_OC_ reconstructions correctly calculate the shifts in *V*_OC_ resulting from differences in the HTL). The presence of a Fermi level drop due to surface recombination can be identified from such DC or CE measurements as a lateral shift of *n* versus *V*_OC_ with contact layers.^[^ ^19^, ^174^ ^]^

*V*_OC_ can also fail to represent the active layer QFLS at high light intensities when the Δ*E*_F_ exceeds the contact built‐in potential.^[^ ^60^, ^66^, ^146^ ^]^ For example, Figure 4b shows the QFLS (Δ*E*_F_) calculated from Equation (3) using TPV and DC data and the measured *V*_OC_ as a function of light intensity for a DP‐PDPP2T‐TT‐based OSC and a Cs_0.17_FA_0.83_Pb(Br_1−_ *_x_* I*_x_*)_3_‐based PSC. It can be seen that both devices show deviations between the calculated QFLS and measured *V*_OC_ at high light intensities, attributed to the pinning of Δ*E*_F_ to the interlayer work functions, as illustrated in Figure 4c.^[^ ^170^, ^174^ ^]^ In the case of such, typically high *V*_OC_, devices, tuning of interlayer work function is critical to optimizing device performance.

The analysis herein focuses on how the energetics of charge accumulation and the kinetics of recombination of this accumulated charge determine *V*_OC_. The charge recombination in this analysis includes both radiative and nonradiative recombination. However, the relatively low photoluminescence quantum yields of almost all OSCs and PSCs indicate that the kinetically dominating recombination pathway limiting *V*_OC_ in these devices is nonradiative. We note that this approach compliments alternative analyses of *V*_OC_ based on measurements of electroluminescence and optical absorption, which allow quantification of the radiative and nonradiative limits to *V*_OC_.^[^ ^159^, ^160^, ^165^, ^175^ ^]^

In summary, for both PSC and OSC, device *V*_OC_ is primarily determined by the effective bandgap of the active layer and the kinetics of charge recombination in the device. Within appropriate limits, and in particular, when charge carriers are mainly in the bulk and *V*_OC_ corresponds to the active layer QFLS, transient photovoltage and differential charging/charge extraction can be used to determine the bimolecular recombination kinetics and dependence of active layer charge density upon QFLS. In this limit, these measurements are direct assays of key properties of the active layer, as we discuss further in the following sections.

### 4.2 Kinetics of Charge Transport and Recombination

An efficient solar cell requires efficient charge extraction from the active layer to the contacts at its maximum power point (i.e., determining device FF). The efficiency of charge extraction in both OSC and PSC is primarily determined by the kinetic competition of charge carrier transport with bulk and surface recombination.

**Figure 6:**
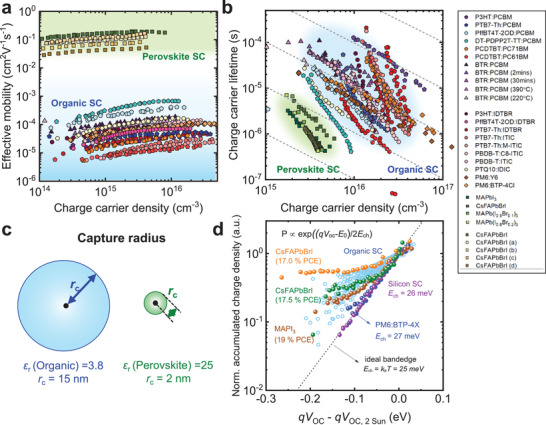
a) The effective mobility as a function of charge carrier density measured at short‐circuit condition and b) the effective charge carrier lifetime *τ_n_* as a function of charge carrier density measured at open‐circuit condition; the dashed lines imply the dependence of charge carrier lifetime versus charge carrier density of ideal bimolecular recombination. c) Schematic illustration of Coulomb capture radius in organic and perovskite semiconductors. The Coulomb capture radius is determined under the condition that the Coulomb attraction potential estimate equals the thermal energy (we note that more conservative estimates equate the Coulomb attraction energy to the magnitude of energy disorder, resulting in significantly smaller capture radius). d) The measured accumulated charge density (normalized at 2 sun illumination condition) versus *qV*_OC_ − *qV*_OC,2sun_ determined from charge extraction measurements at open circuit as a function of irradiation intensity for a range of photoactive layers. The accumulated charge density is normalized to the charge carrier density at 2 sun open‐circuit condition, and the measured *V*_OC_s also plotted relative to these 2 sun values. We note that in the photoactive layer legend, PCBM is PC_71_BM, and CsFAPbBrI (a): Cs_0.17_FA_0.83_Pb(Br_0.23_I_0.77_)_3_, CsFAPbBrI (b): BMP‐doped Cs_0.17_FA_0.83_Pb(Br_0.23_I_0.77_)_3_, CsFAPbBrI (c): Cs_0.17_FA_0.83_Pb(Br_0.1_I_0.9_)_3_, and CsFAPbBrI (d): BMP‐doped Cs_0.17_FA_0.83_Pb(Br_0.1_I_0.9_)_3_. The *J*–*V* performances of the devices shown here were published previously, their fabrication details can be found in the corresponding references.^[^ ^5^, ^19^, ^33^, ^66^, ^78^, ^148^, ^155^ ^]^

We consider first the use of charge extraction at short circuit as an operando assay of the charge transport kinetics in both OSCs and PSCs. This methodology is based on the CE measurement of the charge density in the active layer of the device at short circuit as a function of light intensity (rather than the open‐circuit CE measurements employed in Section 4.1). Under these short‐circuit conditions, bimolecular recombination losses are negligible in most efficient devices. The resultant charge density data can be used to determine effective carrier mobilities (most likely corresponding to the slowest charge carrier^[^ ^176^, ^177^ ^]^). For OSCs, a drift model is employed using the built‐in field from the device contacts.^[^ ^178^ ^]^ For PSCs, due to charge screening by mobile ions, this field is assumed to be negligible, and the carrier mobilities are determined using diffusive charge transport.^[^ ^5^, ^164^ ^]^ Typical effective mobilities for both OSCs and PSCs are shown in **Figure** **6**a. It is apparent that the effective mobilities measured by this CE methodology for typical OSCs range from 10^−5^ to 10^−4^ cm^2^ V^−1^ s^−1^, whereas those measured for PSCs are ≈10^−1^ cm^2^ V^−1^ s^−1^.^[^ ^5^ ^]^ We note that similar mobilities were measured for these devices from microwave conductivity measurements,^[^ ^5^ ^]^ higher bulk mobilities have been reported for some perovskite films, dependent upon film processing and composition.^[^ ^45^ ^]^ This assay thus suggests that the active layer carrier mobilities in PSCs are at least ≈1000‐fold higher than in OSCs.

For both organic and perovskite materials, the quantification of charge carrier mobilities is strongly dependent on the measurement technique employed. For example, terahertz measurements of local, ultrafast carrier motion typically yield higher carrier mobilities than slower timescale CE or microwave assays of bulk mobility, indicative of the greater impact of charge trapping and grain boundaries in the latter.^[^ ^41^, ^179^, ^180^ ^]^ Effective carrier mobilities can also be estimated from the rise kinetics of TPV transients.^[^ ^148^, ^181^ ^]^ We note that the CE assay of effective mobility in PSC devices can be limited by the low mobility of some interlayers;^[^ ^148^ ^]^ as such, the PSC effective mobilities shown in Figure 6a are limited to those obtained for CsFA‐based devices with relatively thin, conductive interlayers, and were found to be in good agreement with those determined for the same active layers by contactless microwave conductivity studies.^[^ ^5^ ^]^ The faster carrier mobilities for perovskites compared to organic semiconductors is in good agreement with both experimental and theoretical literature, and attributed to the dominance of band state transport in PSC and polaron hopping in OSCs,^[^ ^45^ ^]^ as introduced above.

As discussed in the preceding section, for OSC and PSC devices satisfying appropriate conditions (e.g., charge density primarily in the photoactive layer), the TPV and CE/DC charging methodologies can be used to determine the active layer charge carrier lifetime *τ_n_* as a function of charge carrier density *n*, for the recombination mechanism of PSC has been discussed in more depth elsewhere.^[^ ^100^, ^182^ ^]^ This charge carrier lifetime is expected to be primarily limited by bimolecular recombination; as such, in the absence of charge trapping or inhomogeneous charge distributions within the active layer, the lifetime is ideally expected to decrease linearly with increasing charge density (i.e., ideal 2nd order kinetics, indicated as dashed lines in Figure 6b). In practice, for most OSC and PSC devices, the decrease is superlinear, indicative of a reaction order higher than 2,^[^ ^183^ ^]^ attributed primarily to the effect of shallow charge trapping as discussed in the next section (we emphasize that the charge carrier density *n* referred to herein includes all extractable charge, including charges which can be thermally detrapped from shallow trap/tail states). Figure 6b shows typical plots of *τ_n_* versus *n* measured at open circuit for a range of OSCs and PSCs as a function of light intensity, where *τ_n_* is referred to as an effective lifetime, as it includes the effect of reversible, shallow, charge trapping. It is apparent that PSCs exhibit both lower charge densities and shorter carrier lifetimes than OSCs. At matched carrier densities, PSCs exhibit carrier lifetimes 1–2 orders of magnitude faster than OSCs, indicative of bimolecular recombination in the PSC devices studied being 10–100 faster than in OSCs.

The bimolecular recombination times illustrated for OSCs in Figure 6b are in the range 1–100 µs. These lifetimes are much longer the timescale of geminate recombination of charge transfer states generated by exciton separation (typically nanoseconds, see, for example, Figure 2e), consistent with the spatial separation of these charges (≈10 nm for a charge density of 10^18^ cm^−3^) being larger than the size of bound CT states (e.g., Coulomb capture radius ≈ 3–5 nm). In most models of charge recombination in OSCs, bimolecular recombination is considered to result from the collision of two separated charges (one of which may be trapped), which can result in the formation of interfacial CT states, which then either recombine or redissociate.^[^ ^184^, ^185^ ^]^ As such, the properties of these CT states (e.g., how strongly bound they are) are likely to be a key determinant of the kinetics of bimolecular recombination.

For some OSC devices, the charge carrier lifetimes determined by TPV/CE are in good agreement with transient absorption studies of the photoactive layer alone, confirming their assignment to photoactive layer bulk bimolecular recombination. However, for PSCs, demonstrating the equivalence of carrier lifetime measurements from optoelectronic assays such as TPV and optical assays has proved more challenging. In particular, the TPV carrier lifetimes measured for PSCs (10^−7^ to 10^−5^ s dependent upon device and light intensity) are typically slower than equivalent photoluminescence decay times also assigned to bimolecular recombination processes. While the origin of this difference is not fully understood, it most likely results at least in part from the impact of (relatively) nonradiative shallow tail states and/or charge accumulation on charge transport layers. Charge trapping into such tail states can be expected to result in a fast initial PL decay phase, while extending TPV (and potentially residual PL) decay times due to thermally activated detrapping‐mediated bimolecular recombination. Such thermally activated detrapping has, for example, been reported in transient emission studies of copper indium gallium selenide (CIGS).^[^ ^186^ ^]^ In any case, for all devices employed for Figure 4, *V*_OC_ reconstructions using the measured TPV decay times were found to be in excellent agreement with directly measured device *V*_OC_, confirming that the TPV assay of carrier lifetime is a direct determinant of device function.

For most models of bimolecular recombination, the charge carrier lifetime is expected to accelerate linearly with carrier mobility.^[^ ^176^, ^187^, ^188^ ^]^ However, a comparison of Figure 6a,b shows that while the active layer effective mobility of PSCs is ≈10^3^ larger than that of OSCs, the charge carrier lifetimes of PSCs are only tenfold to 100‐fold smaller—corresponding to a larger *μτ* product. This difference is even more striking when one considers that OSC comprises bulk heterojunctions, employed to extend charge carrier lifetimes relative to single‐phase materials. It is thus apparent that PSC photoactive layers exhibit much slower bimolecular recombination kinetics than would be expected for single‐phase organic semiconductors with equivalent carrier mobilities. One likely origin of this difference is the higher dielectric constant (ε_r_ > 25) of perovskite materials relative to organic semiconductor (ε_r_ ≈ 3–4). This results in the Coulomb capture cross‐sectional area for bimolecular recombination being ≈1–2 orders of magnitude lower for perovskite materials, as illustrated in Figure 4c. This means that with a matched charge carrier densities and mobilities, the probability of charge carrier capture and recombination is expected to be 10–100 lower in MAPbI_3_ than in organic semiconductors. This higher dielectric screening in MAPbI_3_ has been attributed to the more ionic nature of this material as well to local ferroelectric effects.^[^ ^163^, ^189^ ^]^ Greater charge carrier delocalization, and lower electron/phonon coupling in MAPbI_3_, as well as photon recycling effects may also contribute to its long carrier lifetimes. In any case, the larger *μτ* product observed for PSC photoactive layers compared to OSC is critical to enabling efficient charge extraction in PSC devices even in the absence of bulk electric fields to drive drift transport, and enabling efficient charge extraction for thicker photoactive layers.

### 4.3 Charge Trapping

For ideal semiconductors, charge carrier mobility is expected to be independent of charge density, and bimolecular recombination lifetimes to decrease linearly with increasing charge density (i.e., ideal 2nd order behavior). However, for both OSC and PSC, such ideal behavior is rarely observed. As illustrated in Figure 6a,b, charge mobilities typically increase with charge density, and charge lifetimes decrease superlinearly with increasing charge density. Such nonideal behavior is typically attributed, at least in part, to shallow charge trapping into tail states in both devices, as illustrated in Figure 6d and discussed in detail in this section.

The presence of sub‐bandgap tail states (also referred to as shallow trap/defect states) in PSC and OSC photoactive layers is measured experimentally by several techniques, including high sensitivity optical absorption measurements,^[^ ^190^ ^]^ photoemission spectroscopies,^[^ ^33^ ^]^ charge extraction,^[^ ^191^ ^]^ and differential charging.^[^ ^66^ ^]^ Charge trapping has been widely reported to have impacts on many aspects of both OSC and PSC functions, including, for example, transport and recombination kinetics,^[^ ^33^, ^66^, ^155^, ^157^ ^]^ and the ideality of *V*_OC_ versus light intensities.^[^ ^5^, ^33^, ^183^ ^]^ In OSCs, the energetic inhomogeneities associated with shallow trap states have been suggested to aid the stabilization of charge separation.^[^ ^192^ ^]^ In most cases, shallow trap states are observed as an exponential density of tail states extending into bandgap (*n* ∝ exp(*E*/*E*_ch_). Figure 6d shows an analysis of the distribution of electronic tail states determined from CE/DC measurements of accumulated charge carrier density as a function of device *V*_OC_ for a range of OSC and PSC devices (these devices exhibited *V*_OC_ ∼ QFLS). For ideal (trap free) band edges, the charge carrier density is expected to follow *n* ∝ exp(*V*_OC_/2*E*_ch_) with *E*_ch_ = *k*_B_ *T* = 25 meV,^[^ ^183^ ^]^ as the electron and hole quasi‐Fermi levels approach the band edges, illustrated as the dashed line in Figure 6d. The charge densities in Figure 6d for OSC and PSC are plotted after normalization to the open‐circuit voltage and charge density at 2 sun (the light intensity for normalization is for convenience only). It is apparent that for most OSCs and PSCs, as the light intensity and *V*_OC_ are reduced, the charge density decreases more slowly than ideal behavior, quantified by a characteristic energy *E*_ch_ > 25 meV and assigned to charges trapped in shallow trap states. For the OSCs and PSCs studied herein, most devices exhibit *E*_ch_ in the range of 40–100 meV, indicative of an energetic distribution of tail states into the bandgap. A notable exception is OSCs employing PBDB‐TF:BTP‐4F as the photoactive layer, which exhibits near‐ideal behavior indicative of almost trap free behavior; we have previously proposed this absence of charge trapping, indicative of the absence of energetic disorder, maybe a key factor between the remarkably high OSC efficiencies reported for this blend.^[^ ^25^ ^]^ We note that the PSCs in this study were limited to PCE in the range <21%; it is likely that very high‐performance PSCs are also likely to exhibit suppressed trap state distributions or densities.^[^ ^193^, ^194^, ^195^, ^196^ ^]^

It is important to note that the trap state densities illustrated in Figure 6d derive from measurements of CE and DC, and so correspond to trapped charges which can be extracted, and therefore contribute to photocurrent. Charges in these tail states undergo thermally activated detrapping, and so can participate in charge transport and bimolecular recombination.^[^ ^197^ ^]^ The mobility and recombination measurements in Figure 6a,b include these shallowly trapped charges. As the charge density in these tail states is increased, the thermal barrier to detrapping from the highest energy trapped carrier decreases, resulting in both the effective carrier mobility and effective bimolecular rate constant increasing with charge carrier density. This shallow charge trapping is therefore distinct from deeper, and therefore effectively irreversible trapping. Such deep trapping can result in monomolecular charge recombination, which has been widely reported in PSCs; for example, the longer charge carrier lifetimes and enhanced PL quantum yields observed with increasing irradiation intensity have been attributed to the filling of these deep traps (for a limited range of light intensities).^[^ ^41^, ^179^, ^180^, ^193^, ^198^, ^199^ ^]^ This contrasts with the impact of increased occupancy of shallow trap states which results, as discussed above, in an acceleration of bimolecular charge recombination and so a decrease in carrier lifetimes. As such, distinguishing between shallow, reversible charge trapping and deep, irreversible charge trapping is critical to determining the impact of trap states on device performance.

The tail state distributions in Figure 6d correspond to nonideal electronic band edges. It is important to note that these are distinct from optical measurements of absorption onsets, often employed to determine optical Urbach energies (see Table 1). In OSCs, such measurements of absorption onset (e.g., high sensitivity external quantum efficiency measurements) probe the homogeneity of the energetics of exciton/CT states, rather than the polaron states probed by CE/DC. Similarly in PSCs, the electrical characteristic energies illustrated in Figure 6d are larger than the corresponding optical (Urbach) energies, potentially resulting from contributions to the electronic data from nonradiative tail states.

While the data in Figure 6d indicate that photoactive layers of both OSC and PSC exhibit exponential tails of shallow trap states with similar ranges of characteristic energies, the densities and physical origins of these tail states differ between these two technologies. There is extensive evidence that PSCs exhibit lower trap densities than OSCs,^[^ ^66^, ^194^, ^196^ ^]^ with, for example, PSC exhibiting a much higher proportion of bimolecular recombination being a radiative band to band recombination. In OSCs, shallow trap states can derive from molecular defects or inhomogeneities (e.g., polymer chain end groups) as well as structural deformations and variations in film crystallinity. As such, molecular design and purification are critical in determining trap densities and energetics in OSCs.^[^ ^33^, ^200^, ^201^, ^202^ ^]^ On the other hand, trap states in PSCs are associated with surfaces and grain boundaries, as well as crystal defects.^[^ ^85^, ^180^, ^203^, ^204^, ^205^, ^206^ ^]^ As such, trap densities and energetics in PSC can be primarily controlled by optimization of film processing, compositional engineering, and surface/grain boundary passivation.^[^ ^77^, ^207^, ^208^ ^]^ Charge trapping in contact layers or at their interface with the photoactive layer can also be important, particularly for PSCs.^[^ ^92^, ^204^, ^209^ ^]^ A suppression of trap state densities in PSC has been widely correlated with increased film photoluminescence, indicative of a suppression of trap‐state‐mediated nonradiative recombination.^[^ ^100^, ^170^ ^]^ In both OSC and PSC, reductions in trap state densities and energies have been correlated with higher device efficiencies, emphasizing the key role of trap states in limiting the performance of both technologies.

## 5 Charge Collection

We turn now to discuss the impact of the kinetic competition between charge transport, bimolecular recombination, and charge collection shown in Figure 3c,d (i.e., with additional external current flow when compared with open‐circuit condition) upon the device *J*–*V* performance. This kinetic competition is critical in determining the efficiency by charges that can be collected as a function of applied bias, and thus determine the shape of the *J*–*V* curve, and the device FF. In particular, when generation current *J*_gen_ is independent of applied bias, the shape of *J*–*V* curve is primarily determined by the increase in bimolecular recombination losses *J*_loss_ due to the increase of charge density as operating condition moves from short circuit toward open circuit, as detailed in the first part in Equation (1) above when then *J*(*V*) ≠ 0, and also in Figure 3.

### 5.1 Device *J*–*V* Reconstruction

**Figure 7:**
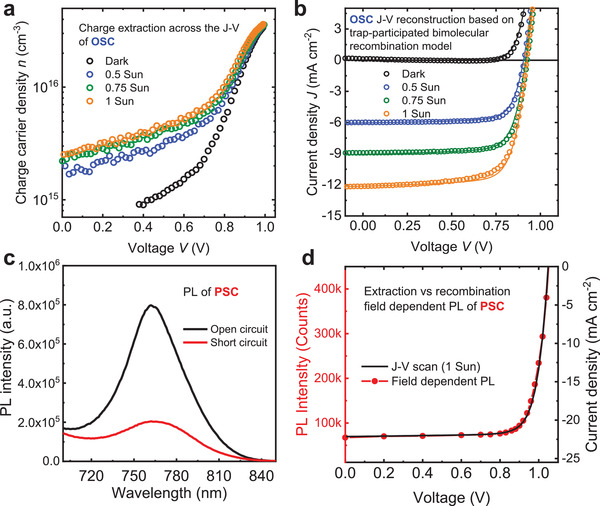
*J*–*V* reconstructions using optoelectrical data for an OSC (BTR:PC_71_BM, see ref. ^[^ ^155^ ^]^ for device fabrication detail) and photoluminescence data for a PSC (MAPbI_3_ device with PTPD HTL). a) The charge extraction data for OSC measured as a function of applied voltage in the dark and under dark, 0.5 sun, 0.75 sun, and 1 sun. b) The measured (lines) and reconstructed (circles) *J*–*V* plots; the reconstruction uses the charge densities *n* from (a) and τ(*n*) from TPV and CE data collected at open circuit as a function of light intensity. c) The PL emission spectra of PSC measured at open circuit and short circuit using 1 sun equivalent irradiation. d) The overlay of measured *J*–*V* and PL emission intensity measured as a function of applied bias.

Following Equation (1), the bimolecular recombination loss current can be probed by transient optoelectronic methods, in particular, the combination of CE measurements at different biases *n*(*V*) and transient photovoltage as a function of charge density measured at open circuit as a function of light intensity *τ_n_*.^[^ ^149^, ^168^, ^210^ ^]^ This methodology exploits the potential of CE measurements to determine the charge density across the *J*–*V* curve rather than just at open or short circuit. We note that this calculation uses of *J*_loss_(*V*) uses *τ_n_* measured at open circuit to calculate carrier lifetimes across the *J*–*V* curve, and so assumes that *J*_loss_ is only a function of *n*, neglecting, for example, possible differences in the spatial distribution of charge as a function of voltage. Despite the severity of this assumption, it is remarkable that, at least for OSC, this methodology is able to reconstruct the dark and light *J*–*V* curves of many devices.^[^ ^149^, ^168^, ^210^ ^]^ It should be noted that such reconstructions require relatively large data sets, and so are only possible with particularly stable OSCs. Typical data for stable field‐independent generation OSC devices are illustrated in **Figure** **7**, with Figure 7a showing charge extraction data as a function of applied bias, and Figure 7b the corresponding reconstructed *J*–*V* using these charge extraction data. It is apparent that an accurate reconstruction is obtained both in the light and dark, clearly demonstrating the validity of the simple device model introduced in Section 4 in determining not only the *V*_OC_ but also the FF of OSCs.

The *J*–*V* reconstruction for an OSC illustrated in Figure 7b uses charge extraction data to determine charge density. As discussed above, CE measurements on PSCs typically give erroneous charge densities, most likely due to ion motion in the device, and so this approach cannot be applied to PSCs.^[^ ^211^ ^]^ However for PSCs, photoluminescence provides an alternative probe of the bimolecular recombination flux in the device (for OSCs, most PL derives from exciton decay, and so cannot normally be used as a probe of bimolecular recombination). Figure 7c shows an example of PL emission of a PSC device measured at open circuit and short circuit under 1 sun equivalent irradiation; the quenching of PL intensity from open to short circuit reflects suppression of bimolecular recombination losses due to charge extraction at short circuit. The efficiency of this quenching of PL between open and short circuit can be used as an assay of the extraction efficiency of the device.^[^ ^148^, ^166^ ^]^ In addition, measurement of this PL intensity as a function of bias allows measurement of the voltage dependence of bimolecular recombination, *J*_loss_ and thus, following Equation (1), a reconstruction of the device *J*–*V* curve.^[^ ^102^ ^]^ A typical *J*–*V* reconstruction using this approach is illustrated in Figure 7d. We note that this PL‐based *J*–*V* reconstruction is only possible if the relative proportions of radiative and nonradiative recombination in the device are independent of applied bias; for example, this approach works less well for PSCs with highly doped HTLs such as PEDOT:PSS which exhibit severe nonradiative surface recombination.^[^ ^19^, ^92^ ^]^ Nevertheless, the success of *J*–*V* reconstructions such as those shown in Figure 7b,d demonstrates the importance of bimolecular recombination in determining the fill factor of many efficient OSC and PSC devices.

For both OSC and PSC devices, bimolecular recombination losses become a less severe limitation on the efficiency of charge extraction at lower light levels, due to the lower densities of accumulated charge carriers.^[^ ^155^ ^]^ As such, for both technologies, device efficiencies tend to increase at lower light intensities, in direct contrast to efficiency decreases typically observed for silicon devices, important for applications such as indoor energy harvesting.

### 5.2 Impact of Charge Transport Layers

Charge transport layers are critical to the optimization of both OSC and PSC performances. The conductivity of the transport layers must be sufficient to avoid resistive losses, and their energetics aligned to the energetics of the photoactive layer.^[^ ^170^, ^174^ ^]^ This energetic requirement is important for both OSC and PSC. The selectivity of the transport layer for either electron or hole extraction is also important, particularly for PSCs, as we discuss further in this section.

**Figure 8:**
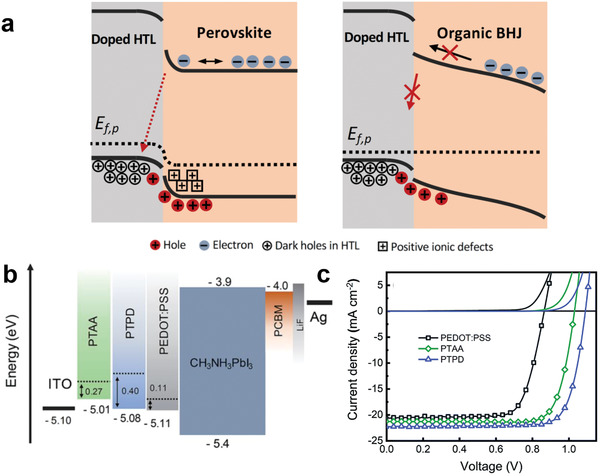
a) Schematic drawing of flat‐band energy diagram of a series of p–i–n PSCs with different organic HTLs, PEDOT:PSS, PTAA, and PTPD, including their HOMO and equilibrium Fermi levels. b) Schematic drawings of open‐circuit band diagram and charge/ion accumulation at the photoactive layer/doped HTL interface for PSCs and OSCs. c) *J*–*V* curves of PSCs under AM1.5 1 sun equivalent illumination at a scan rate of 50 mV s^−1^ in reverse bias. a–c) Reproduced with permission.^[^ ^19^ ^]^ Copyright 2019, Royal Society of Chemistry.

Transport layer optimization appears to be more critical for PSC than OSC. PSC charge transport layers can influence the crystallization of the perovskite active layer and/or passivate surface perovskite traps.^[^ ^19^, ^212^, ^213^, ^214^ ^]^ Optimization of the transport layer is also important to minimize nonradiative surface recombination at the perovskite/charge transport layer interface, which can result in a loss of QFLS near this interface and thus lower device *V*_OC_s.^[^ ^215^, ^216^, ^217^, ^218^, ^219^ ^]^ For example, while PEDOT:PSS has been found to work well as a HTL in p–i–n OSCs, and indeed is often employed in high performance devices, in PSCs, it has been found to result in excessive surface recombination losses, and relatively poor performance. As a consequence, there is extensive work on alternative HTLs for p–i–n PSCs. An example of such a study is illustrated in **Figure** **8** for PSCs employing either PEDOT:PSS or two alternative organic semiconductors as HTLs.^[^ ^19^ ^]^ For this series, the device performance was found to correlate most clearly with the doping density of the HTL (determined from measurement of the HTL work function relative to its HOMO level), rather than the HOMO level alone. Insensitivity of *V*_OC_ to HOMO level has been observed for a variety of HTLs in PSCs.^[^ ^220^, ^221^, ^222^ ^]^ High HTL doping was suggested to result in excessive surface recombination losses, resulting in a loss of QFLS at the HTL/perovskite contact.^[^ ^19^, ^176^ ^]^ It is striking that high HTL doping (such as for PEDOT:PSS) can result in excessive surface recombination losses in PSCs but not OSCs. A possible reason for this is illustrated in Figure 8c. OSCs exhibit strong built‐in electric fields generated by differences in electrode work function, which drive electrons and holes to the electron‐transport layer (ETL) and HTL layers, respectively. By contrast, in PSCs, this electric field is screened by ion migration in the bulk perovskite, with charge transport resulting primarily from diffusion. As such, the requirements for selective HTL/ETL contacts may be more severe for PSCs than OSCs, with a range of strategies being employed to achieve more selective extraction and minimize surface recombination in PSC devices.^[^ ^176^, ^177^, ^178^, ^179^, ^180^, ^181^, ^182^, ^183^, ^184^, ^185^, ^186^, ^187^, ^188^, ^189^, ^190^, ^191^, ^192^, ^193^, ^194^, ^195^, ^196^, ^197^, ^198^, ^199^, ^200^, ^201^, ^202^, ^203^, ^204^, ^205^, ^206^, ^207^, ^208^, ^209^, ^210^, ^211^, ^212^, ^213^, ^214^, ^215^, ^216^, ^217^, ^218^, ^219^, ^220^, ^221^, ^222^, ^223^, ^224^ ^]^

### 5.3 Photoactive Layer Thickness Dependence

As discussed in Section 5, the efficiency of charge extraction to the external circuit is primarily determined by the kinetic competition between charge transport and bimolecular recombination. This efficiency of charge collection is often quantified by the photoactive layer *μτ* product. The *μτ* product is critical in determining the dependence of the extraction efficiency upon photoactive thickness. A larger *µτ* product is associated with a longer transport length, and also normally results in a higher device FF.^[^ ^225^, ^226^, ^227^, ^228^ ^]^ For OSCs, the optimum thickness of most photoactive layers is typically ≤100 nm for efficient device performance (there are exceptions such as DT‐DPP2T‐TT:PC_71_BM and BTR:PC_71_BM (BTR = 5,5′‐ [[4,8‐bis[5‐(2‐ethylhexyl)‐4‐hexyl‐2‐thienyl]benzo[1,2‐b:4,5‐b′]dithiophene‐2,6‐diyl]bis[(3′,3″‐dihexyl[2,2′:5′,2″‐terthiophene]‐5″,5‐diyl)methylidyne ]]bis[3‐hexyl‐2‐thioxo‐4‐thiazolidinone]), which show high performance with thicker photoactive layers, as discussed below), while PSCs exhibit efficient performance for photoactive layers over 1/2 µm thick; this is a particular challenge for OSC commercialization due to the difficulty of homogeneously printing such thin layers over large areas. This difference in behavior between OSC and PSC devices primarily results from the much higher mobility of perovskite light absorbers compared to most organic semiconductor blends, as discussed in Section 4.

**Figure 9:**
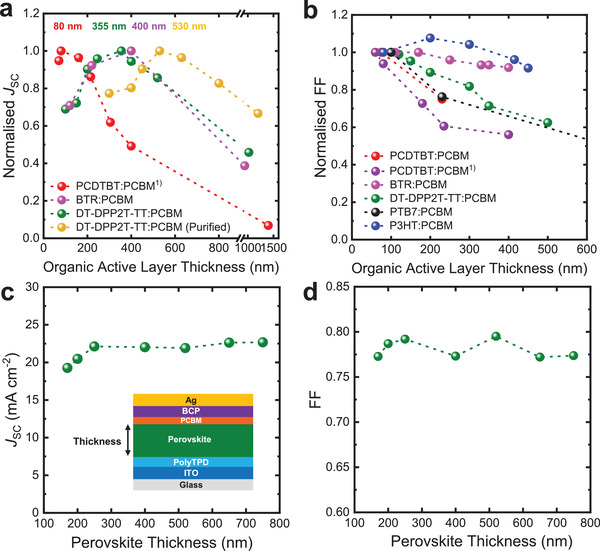
a) Current densities (*J*_SC_) and b) fill factors (FF) of a selection of organic solar cells plotted as a function of the photoactive layer thickness.^[^ ^66^ ^]^ c) *J*_SC_ and d) FF of MAPbI_3_ solar cells as a function of perovskite film thickness.^[^ ^78^ ^]^ We note that "(purified)" in the legend in (a) means purified DT‐DPP2T‐TT donor (we note that PCBMs in the figures are PC_71_BM, and PCBM^1)^ is PC_61_BM). a,b) Reproduced under the terms of the CC‐BY Creative Commons Attribution 4.0 International license (https://creativecommons.org/licenses/by/4.0).^[^ ^66^ ^]^ Copyright 2019, The Authors, published by Springer Nature. c,d) Reproduced with permission.^[^ ^78^ ^]^ Copyright 2020, Royal Society of Chemistry.

Typical data for both OSC and PSC devices, plotting *J*_SC_ and FF as a function of photoactive layer thickness are plotted in **Figure** **9**. For both PSC and OSC, increases in current result from enhanced light absorption.^[^ ^229^, ^230^ ^]^ For PSCs, both *J*_SC_ and FF are typically observed to be relatively insensitive to photoactive layer thickness, attributed to their larger *μτ* product, with estimates of charge diffusion transport lengths often exceeding 1 µm^[^ ^77^, ^95^, ^194^ ^]^ (it should be noted that determination of both μ and τ depend on measurement procedure, and for τ in particular on charge carrier density). For OSCs, both *J*_SC_ and FF show stronger dependencies on photoactive layer thickness, as illustrated in Figure 9. For most OSCs, device FF drops off for photoactive layer thickness greater than 100 nm. However, there are exceptions, with some organic blend systems showing thickness‐insensitive FF up to several hundred nanometers.^[^ ^231^, ^232^, ^233^ ^]^ These organic systems are often referred to as blends exhibiting non‐Langevin bimolecular recombination (this term refers to systems exhibiting slower recombination than that predicted from mobility measurements using Langevin recombination theory).^[^ ^234^ ^]^ For example, for the blends shown in Figure 9b, P3HT:PC_61_BM and BTR:PC_71_BM blends maintain high FF with thicker photoactive layer and show non‐Langevin factors of ≈10^−3^ (i.e., bimolecular recombination rate constants 1000 slower than that expected for Langevin behavior). By contrast, the other blends, which exhibit drops in FF with increasing thickness, show non‐Langevin factors of ≈10^−1^. Non‐Langevin blend systems have been suggested to result, at least in part, from favorable blend morphologies, with phase segregation spatially separating electrons and holes while maintaining pathways for efficient charge transport.^[^ ^177^, ^234^, ^235^ ^]^ For comparison only, an equivalent non‐Langevin factor for a typical PSC would be of the order of 10^−2^ to 10^−3^. Many OSCs also exhibit a loss of *J*_SC_ for thicker films, often associated with a formation of a space charge layer which screens the internal electric field. Such space charge layer formation has been attributed to unintentional doping,^[^ ^236^, ^237^ ^]^ mobility mismatch,^[^ ^225^ ^]^ and/or photogenerated charge accumulation into tail states.^[^ ^66^ ^]^ In summary, it is apparent that higher carrier mobilities, and higher *μτ* products of perovskite light absorbers relative to most organic blends enable efficient charge extraction in thick PSCs via diffusion alone, even in the absence of bulk electric fields to drive drift transport. Achieving efficient charge extraction in OSCs with active layer thicknesses commensurates with large area printing requirements, with notable promising exceptions,^[^ ^232^, ^237^ ^]^ a key challenge of OSC research.

## 6 Summary and Future Perspectives

Herein, we focus on the charge carrier dynamics in p–i–n organic and perovskite solar cells, and how their similarities and differences in design requirements and function result from underlying material properties. The organic semiconductors employed in the photoactive layer of OSCs are relatively soft materials, with a low dielectric constant, resulting in Frenkel exciton states, relatively low charge carrier mobilities associated with polaron formation and charge trapping. A key challenge for these devices is the efficient separation of Coulombically bound exciton and charge transfer states to yield dissociated charges, typically driven by donor/acceptor bulk heterojunctions. Charge collection in OSCs requires built‐in electric fields to drive charge extraction through drift transport. Key material challenges for these materials include control of blend nanomorphology, optimization (and minimization) of heterojunction energy offsets, minimization of trap state densities and distribution, and increase of effective charge carrier mobilities. On the other hand, the perovskite absorber layer in PSCs is relatively crystalline with a higher dielectric constant, resulting in photoexcitation directly generating separated charges. Due to the presence of mobile ions which screen bulk electric fields, charge transport in PSCs is primarily by diffusion rather than by drift. Nevertheless, the much higher carrier mobilities and lower trap state densities can enable relatively fast and efficient charge extraction. Key performance limitations are recombination losses mediated by charge trapping, particularly at grain boundaries, and at interfaces with charge extraction layers.

OSC and PSC differ qualitatively in their primary photophysics, with the requirement for exciton and charge transfer state dissociation on ultrafast timescales being a key determinant of the efficiency of photocurrent generation in OSCs. There is however increasing evidence that with the selection of appropriate materials, this requirement for exciton/charge transfer state dissociation may not impose a fundamental limit on the efficiency of OSCs relative to PSCs. By contrast, in terms of device fill factor and open‐circuit voltage, OSCs and PSCs exhibit strong similarities. In both devices, *V*_OC_ is driven by photoinduced QFLS in the bulk, with the active layer electronic bandgap and recombination kinetics of accumulating charges being the primary determinants of its magnitude for a given light intensity. Similarly in both devices, FF is often primarily determined by the kinetic competition charge transport and recombination which determines the efficiency of charge extraction. More quantitatively, the faster carrier mobilities, lower trap densities, and only moderately fast recombination kinetics observed for PSCs relative to OSCs result in typically higher *V*_OC_s (for matched optical bandgaps) and efficient charge extraction from thicker photoactive layers. For both devices, charge trapping is a key limitation on efficiency, although the material origins of these traps differ. Optimization of charge extraction layers is important to achieve high performance for both technologies, but appears to be the most critical for PSCs, which have more severe requirements on contact selectivity.

PSCs currently exhibit higher state‐of‐the‐art efficiencies than OSCs, although OSC efficiencies are increasing rapidly at present. In terms of fundamental limits to the device efficiency, the relatively soft nature of organic semiconductors, manifested as enhanced electron/phonon coupling, greater nonradiative recombination, and energetic losses associated with polaron formation, may, depending on material design, continue to limit the maximum achievable efficiencies for OSCs. On the other hand, OSCs have some advantages over PSCs, such as spectral tunability (e.g., for window applications) and lower levels of concern over material toxicity. Further key challenges, not covered in this perspective, include stability and manufacturability. We hope that this Perspective has provided some useful insights into the similarities and differences of the photophysics and charge carrier dynamics underlying the function of organic and perovskite solar cells which will help to guide further advances in materials and device performance.

## Conflict of Interest

The authors declare no conflict of interest.
